# Subthalamic Nucleus Deep Brain Stimulation Impacts Language in Early Parkinson's Disease

**DOI:** 10.1371/journal.pone.0042829

**Published:** 2012-08-07

**Authors:** Lara Phillips, Kaitlyn A. Litcofsky, Michael Pelster, Matthew Gelfand, Michael T. Ullman, P. David Charles

**Affiliations:** 1 Department of Neurology, Vanderbilt University, Nashville, Tennessee, United States of America; 2 Brain and Language Lab, Department of Neuroscience, Georgetown University, Washington, District of Columbia, United States of America; 3 Department of Psychology, Center for Language Science, Pennsylvania State University, University Park, Pennsylvania, United States of America; INSERM/CNRS, France

## Abstract

Although deep brain stimulation (DBS) of the basal ganglia improves motor outcomes in Parkinson's disease (PD), its effects on cognition, including language, remain unclear. This study examined the impact of subthalamic nucleus (STN) DBS on two fundamental capacities of language, grammatical and lexical functions. These functions were tested with the production of regular and irregular past-tenses, which contrast aspects of grammatical (regulars) and lexical (irregulars) processing while controlling for multiple potentially confounding factors. Aspects of the motor system were tested by contrasting the naming of manipulated (motor) and non-manipulated (non-motor) objects. Performance was compared between healthy controls and early-stage PD patients treated with either DBS/medications or medications alone. Patients were assessed on and off treatment, with controls following a parallel testing schedule. STN-DBS improved naming of manipulated (motor) but not non-manipulated (non-motor) objects, as compared to both controls and patients with just medications, who did not differ from each other across assessment sessions. In contrast, STN-DBS led to *worse* performance at regulars (grammar) but not irregulars (lexicon), as compared to the other two subject groups, who again did not differ. The results suggest that STN-DBS negatively impacts language in early PD, but may be specific in depressing aspects of grammatical and not lexical processing. The finding that STN-DBS affects both motor and grammar (but not lexical) functions strengthens the view that both depend on basal ganglia circuitry, although the mechanisms for its differential impact on the two (improved motor, impaired grammar) remain to be elucidated.

## Introduction

Parkinson's disease (PD), the second most common neurodegenerative disorder in America [1], is characterized by tremor, rigidity, bradykinesia, and postural instability. Although it is generally thought of as a motor disorder, PD also affects cognition, including language [2]–[5]. However, these impairments are less well characterized than the motor deficits.

PD symptoms develop after degeneration of dopaminergic neurons that project from the substantia nigra pars compacta to the striatum, ultimately decreasing thalamic excitation of motor and other cortical areas [6]. PD symptoms can be largely controlled with dopaminergic medications. However, these medications have various adverse side effects. Furthermore, as the disease progresses, the efficacy of the medications diminishes, increasing their daily dose requirements. Due in part to these problems, interest has increased in alternative treatments such as deep brain stimulation (DBS). DBS is not associated with the side effects of dopaminergic medications, and appears to be superior to optimal drug therapy for ameliorating motor function and quality of life in patients with advanced PD [7]. DBS improves motor function in PD patients by normalizing basal ganglia-thalamic output to motor cortex. The mechanisms of DBS are still not well understood, but it appears to lead to network-wide changes of neural activity, modulated by various neurotransmitters [8], [9].

Here, we examine the impact of subthalamic nucleus (STN) DBS on language, in particular on two fundamental aspects of language: (1) the mental lexicon, which is the mental dictionary of memorized words and their meanings; and (2) the mental grammar, which underlies the rule-governed sequential and hierarchical composition of complex forms, including in phrases and sentences (syntax; e.g., *the*+*cat*) and complex words (morphology; e.g., in regular past-tense formation, such as *walk*+*-ed*). Evidence suggests that these two capacities depend on largely distinct neural and computational bases [10]–[12]. The mental grammar depends in part on frontal/basal-ganglia circuits responsible for procedural memory, which subserves motor and cognitive skills; the mental lexicon depends on a largely different network [10], [13]. Consistent with this neurocognitive dichotomy, the two capacities are differentially affected in PD. Grammatical processing is often, though not always, impaired, across syntax [14]–[16] and morphology (e.g., in the production of regular past-tenses) [3], [17], but see [18]. In contrast, lexical knowledge appears to remain relatively spared [19], [20], though retrieval of words can be problematic [21].

Few studies have examined the impact of STN-DBS on lexical and grammatical functions in PD. Lexical knowledge and retrieval have shown inconsistent patterns in response to STN-DBS, variously improving [22], [23], worsening [23], [24], or showing no changes [22], [25]. The effect of STN-DBS on grammatical processing has been examined in only two studies, which reported improvements in some but not other measures of both syntax and morphology [26], [27].

Thus, the literature examining the impact of STN-DBS on lexical and grammatical processing is still quite small, and has elicited inconsistent results. This inconsistency is likely due to various factors – including not only the variability of lexical and grammatical measures, but also the difficulty in selecting adequate tasks and control conditions. For example, negative effects of STN-DBS on verbal fluency [28] could be due to various impairments, including of retrieval, search, and executive functions. This suggests caution in drawing conclusions as to whether previously-observed STN-DBS effects were due to changes in actual lexical or grammatical processing, or to confounding factors such as motivation, attention, or even motor function (resulting in changes in manual or oral responses). Together with the importance of intact language for the quality of life of PD patients, this suggests the need for further research to elucidate the impact of STN-DBS on this critical cognitive domain.

The current study investigates the impact of STN-DBS on lexical and grammatical processing in a well-controlled and well-studied language paradigm: the production of past-tense forms of regular and irregular verbs [3], [29], [30]. Irregular past-tenses (e.g., *dug*), being idiosyncratic, must depend on memorized lexical representations, which are retrieved during past-tense production. Regular past-tenses are rule-governed complex words (add *–ed* to the stem) that can be composed from their parts (e,g, *walk*+*-ed*) by the mental grammar. Regulars and irregulars can be matched, directly or statistically, on multiple factors, such as word frequency and phonological complexity. Thus, lexical and grammatical processing can be contrasted in the same task, allowing one to examine effects that are specific to one versus the other type of processing, while controlling for a wide range of potentially confounding factors (including subject-level variables such as attention and motivation, which would be expected to similarly affect the two conditions).

Although the past-tense production task allows us to isolate any STN-DBS related changes specific to lexical or grammatical processing, it would also be highly informative to examine the relation between any such changes in language and STN-DBS related changes in the motor system. Previous STN-DBS studies have probed motor function with tasks that examine physical motor outcomes [31]. Although this has the advantage of directly testing the outcome of interest, the observed improvements could be explained by confounding factors such as motivation or attention. In addition, with physical motor tasks it can be unclear whether motor impairments or improvements are due to changes in low-level problems such as tremor, or higher-level motor knowledge or processing. To address these issues, we examined one aspect of motor-related outcomes of STN-DBS, that is, higher-level motor knowledge or processing, with a task that directly contrasts this knowledge/processing with a matched control condition. Subjects are asked to name pictures of objects that either are or are not commonly manipulated (e.g., *hammer*, *elephant*). Evidence suggests that naming manipulated objects (e.g., tools and utensils), but not non-manipulated objects, critically depends on motor-related circuits, and that damage to these circuits can impair their processing, including their naming [32]–[39]. Since subjects perform the same task for both manipulated and non-manipulated objects, which are moreover matched on various factors (e.g., word frequency and word length), a relative change in naming manipulated versus non-manipulated objects suggests changes specific to aspects of higher-level motor knowledge or processing. Thus, this object naming task parallels the past-tense production task, in which lexical and grammatical functioning are directly contrasted, allowing effects in each to be identified.

This study examined language and motor domains with past-tense production and object naming tasks in early PD patients enrolled in a clinical trial of STN-DBS at Vanderbilt University. Three groups of subjects were tested: patients being treated with STN-DBS (and optimal drug therapy, as in previous studies of STN-DBS in PD, including in all the studies discussed above), patients being treated only with optimal drug therapy, and healthy control subjects. The examination not just of patients with STN-DBS, but also patients taking medications alone, allows the effects of STN-DBS to be compared with the effects of optimal medical treatment. All subjects were tested twice. Patients were tested first on and then off all treatments, with a five day washout period between the two test sessions. Testing of control subjects followed the same schedule. The validity of the off state was ensured by eliminating both stimulation and medication after the first test session, together with the long washout period, since the effects of medications and DBS may last hours to days [40], [41]. Note that such a long washout period is possible with early PD patients, whereas more advanced PD patients are unlikely to be able to tolerate such an extended lack of treatment.

Since previous evidence from patients with more advanced PD suggests a positive impact of STN-DBS on motor functioning, even as compared to optimal drug therapy, we expected a similar pattern in patients with early PD, as evidenced by STN-DBS-related improvements in naming manipulated objects relative to non-manipulated objects. We also expected an improvement of grammar (regulars), since (1) STN-DBS is targeted at and improves motor function; (2) evidence suggests common neural substrates (procedural memory) for grammar and motor skills (see above); and (3) the only two studies examining the effects of STN-DBS on grammar reported improvements in advanced PD. Note that since language and other cognitive problems tend to appear during later stages of PD, early PD patients might not show any obvious difficulties with grammar, and thus might not show room for improvement. To address this possibility we acquired (for both tasks) not just accuracy as a dependent measure, but also response times, which are less susceptible to ceiling effects. This increases the likelihood of observing not only any existing PD deficit and/or STN-DBS related improvement, but also any STN-DBS related decline in performance. Finally, given the lack of common neural substrates for lexical and motor functions, and the previous inconsistent outcomes of STN-DBS on tasks probing lexical functions, we did not predict any STN-DBS changes for irregulars.

## Methods

### Subjects and Protocol

The PD patients in this study were drawn from participants enrolled in a pilot clinical trial, “Safety and Tolerability of Deep Brain Stimulation in Early Stage Parkinson's Disease” (NCT00282152), which examines STN-DBS in early stage PD at Vanderbilt University Medical Center. In this pilot clinical trial, the PD patients were initially randomized to receive either DBS surgery and optimal drug therapy (“DBS+ODT patients”) or only optimal drug therapy (“ODT patients”), using block randomization to ensure equal numbers of patients within the two groups. Patients were required to meet a number of criteria in the pilot clinical trial (see Table 1). The pilot was conducted with an investigational device exemption from the US Food and Drug Administration (G050016) and was approved by the Vanderbilt University IRB (040797), in accordance with the Declaration of Helsinki.

**Table 1:** Inclusion and exclusion criteria.

Inclusion Criteria	Exclusion Criteria
Parkinson's Disease patients	
• Response to dopaminergic therapy	• Evidence of an alternative diagnosis or secondary parkinsonism
• Hoehn and Yahr stage II when off medication	• Uncontrolled medical condition or clinically significant medical disease that would increase the risk of developing pre- or post-operative complications (e.g., significant cardiac or pulmonary disease, uncontrolled hypertension)
• No contra-indications to surgery	• Dementia
• Aged 50–75 years	• Major psychiatric disorder
• MRI deemed normal for their age	• Previous brain operation or injury
• Have taken levodopa or dopamine agonist therapy for greater than or equal to six months, but less than or equal to four years	• Active participation in another clinical trial for the treatment of PD
	• Cardiac pacemakers or medical conditions that preclude MRI scans
	• Evidence of existing dyskinesias or motor fluctuations
Control subjects	
• Aged 50–75 years	• Dementia
	• Major psychiatric disorder
	• Brain operation or injury
	• Parkinson's Disease

The DBS surgical procedure targets the subthalamic nucleus bilaterally, with the goal of treating motor symptoms. The subthalamic nuclei were identified using standard stereotactic procedures and mapping techniques. Areas surrounding the STN involved in speech and articulation were avoided, on the basis of speech and articulatory tasks performed during the procedure (in order to avoid dysarthria or secondary motor dysfunction due to disruption of nearby white matter tracts). Bilateral leads and pulse generators were subsequently implanted. The stimulators were programmed at a high frequency (130 Hz with a pulse width of 60 microseconds). During treatment, stimulators are used 24 hours/day.

According to the protocol of the pilot clinical trial, patients returned for follow-up inpatient visits every six months for two years after DBS implantation (or after only baseline assessment for ODT patients). The present study examined all patients who were available during these visits, from May 2008 through March 2009: 11 DBS+ODT and 11 ODT patients. The number of months from DBS implantation or baseline to testing did not differ between the DBS+ODT and the ODT groups (Table 2). The L-dopa equivalent dose at time of testing also did not differ significantly between the two groups, though, as expected, the dose was slightly (marginally significantly) higher in the ODT group than in DBS+ODT group (Table 2). All patients in this study were monolingual native speakers of English. Because all but one patient in each of the two groups were male, and evidence suggests sex differences in the neurocognition of regulars, and of grammar more generally [29], [42], we excluded the two females from analyses.

**Table 2:** Participant demographic and other information.

	Controls	DBS+ODT	ODT	Comparison
Age (years)	56.9 (*6.0*)	61.4 (*6.2*)	59.7 (*5.6*)	*F*(2, 37) = 2.0, *p* = .150
Education (years)	15.4 (*2.4*)	15.8 (*2.9*)	14.4 (*1.6*)	*F*(2, 37) = 0.96, *p* = .391
Handedness	62.6 (*55.8*)	83.1 (*24.9*)	49.4 (*59.8*)	*F*(2, 37) = 1.12, *p* = .337
Time from DBS implantation or baseline to testing (months)	N/A	13.8 (*6.4*)	11.4 (*6.0*)	*t*(18) = 0.87, *p* = .395
L-dopa equivalent dose (mg)	N/A	390.7 (*246.5*)	645.4 (*343.5*)	*t*(18) = 1.91, *p* = .073

Means are presented, with standard deviations in parentheses. Education reflects years of schooling starting from first grade. Handedness reflects laterality quotients from the Edinburgh Handedness Inventory [47], where 100 represents strongly right-handed and −100 represents strongly left-handed. Comparisons reflect one-way ANOVAs across the three groups (for Age, Education, and Handedness), otherwise an independent-samples *t*-test between the DBS+ODT and ODT groups.

Twenty-one healthy control subjects (“controls”) were recruited (see Table 1). All were monolingual speakers of English. To ensure that they were sex-matched to the PD patients, three female controls were excluded; analyses were performed on the remaining 18 male controls. The control subjects were matched on age, education, and handedness to the DBS+ODT and ODT groups (see Table 2).

During the inpatient visit, patients were tested on Day 1 (Session 1; on treatment), and, after a five day washout, again on Day 7 (Session 2; off treatment), on both the past-tense production task and the object naming task (with that task order). Control subjects were tested according to the same schedule.

IRB approval for the current study was acquired from Vanderbilt University (080302), in accordance with the Declaration of Helsinki. Written informed consent was obtained from all subjects (patients and controls) prior to testing.

### Tasks

Details on both tasks have been previously reported [3], [29], [30]. In brief, in past-tense production subjects are asked to produce out loud past-tenses of regular and irregular verbs, given their visually-presented stems. In object naming, subjects are asked to name out loud pictures of commonly manipulated (“manipulated”) objects and objects that are not commonly manipulated (“non-manipulated”). Two versions of each task were prepared; each version of each task contained distinct items. Each subject received different task versions in the two test sessions (counterbalanced across subjects), thus avoiding item repetition between the two sessions.

#### Past Tense Production Task

Each of the two versions of the task contained 72 verbs: 20 regular verbs (e.g., *fail-failed*), 20 irregulars (e.g., *hold-held*), and 32 filler verbs. See Table 3. The first 20 regular and first 20 irregular verbs listed in Table 3 are from one version, while the remaining 20 of each are from the other version. The regular and irregular verbs were matched statistically to each other and between the two task versions on a wide range of factors by including these factors as covariates in the analysis model (See Data Analysis). The regulars were all “consistent” regulars, whose stems are phonologically dissimilar to the stems of irregulars (e.g., *glide-glided*, cf. *hide-hid*, *ride-rode*, was excluded) [29]. The irregulars did not include any no-change verbs (e.g. *hit-hit*). We attempted to avoid doublet verbs, i.e., those that can take both a regular and an irregular past-tense (e.g., *dive-dived*/*dove*) [29]. All regulars and irregulars were monosyllabic in their stem and past-tense forms. The filler verbs, not discussed further here, included (in each of the two versions) 10 “inconsistent” regulars (e.g., *squeeze-squeezed*), whose stems are phonologically similar to the stems of irregulars, and whose past-tenses have an increased likelihood of storage; 6 “-*ed* plausible” irregulars whose past-tenses are plausible –*ed*-affixations of stems that have undergone vowel changes (e.g., *sweep-swept, flee*-*fled*); and 16 novel verbs (e.g., *crog*) [29], [43]. Verbs were pseudo-randomized, separately for each version, such that there were not more than 3 consecutive verbs of any type, nor strings of more than 3 regulars (consistent or inconsistent) without an intervening irregular or novel verb. To avoid priming effects, verbs with similar sounding forms (e.g., *fling, tring*) never appeared in adjacent positions. Moreover, there were at least 5 intervening verbs between any two verbs whose stems or past tense forms rhyme. In no case were there more than two verbs in a row with the same stem vowel, or with the same past-tense vowel.

**Table 3:** Past-tense production task: items.

**Regular verbs**	chew, cry, drop, fail, gain, guess, hope, hurl, knock, pull, push, save, slash, smooth, snoop, spray, stop, talk, try, wish, blame, cause, claim, droop, flog, gulp, lurk, pose, pray, sigh, slip, spur, stay, stir, tie, view, vow, watch, weigh, work
**Irregular verbs**	bend, catch, choose, deal, drive, feed, fight, freeze, grow, lend, ride, shake, shoot, slay, steal, stick, sting, string, swear, swing, bleed, blow, breed, build, dig, draw, feel, fling, hide, hold, meet, run, sling, speak, spend, teach, throw, wear, win, write

Stimuli were visually presented on the CRT screen of a PC computer using E-Prime 1.2. Subjects were instructed to change the word according to the model “*eat→ate*”, such that the new word would fit in the prompt sentence *Yesterday, I _______*. Subjects were requested to respond as quickly and accurately as possible. The instructions included a number of additional verb examples, for which feedback was provided to the subject. Subjects were then given 6 practice items, which were presented in the same manner as the subsequent test items. To acclimate the subject to the task and to minimize early item order effects, the test sequence itself began with 9 additional items (not counted in the 72 described above), which were excluded from analysis. Presentation of the verb stem initiated an E-Prime timer, which was terminated by the subject's oral response (triggered via a microphone connected to the computer). Response times were defined as the difference between the onset of presentation of the verb stem and the onset of the oral response. The verb stem and prompt sentence remained on the screen up to 10 seconds, or until the experimenter pressed a mouse button after the subject responded. Subsequently, a 2770 ms Inter-Stimulus Interval (ISI) was presented (200 ms advance tone, 1800 ms blank screen, 750 ms fixation cross, 20 ms blank screen), followed by the next item. The entire session was audio-recorded. Responses were transcribed phonemically by two trained transcribers: the experimenter, who transcribed responses during testing, and an independent transcriber, who transcribed from the recording. The rare disagreements were resolved by a third trained transcriber.

#### Object Naming Task

Each of the two versions of this task consisted of 64 color pictures of objects (see Table 4). Thirty-two in each version were man-made objects that are commonly manipulated or otherwise physically interacted with, including tools and utensils (“manipulated” objects; e.g., *hammer, umbrella*). The other 32 were animals that are not commonly manipulated or physically interacted with (“non-manipulated” objects; e.g., *lion, scorpion*). The first 32 manipulated objects and first 32 non-manipulated objects listed in Table 4 are from one of the two task versions, while the remaining 32 of each are from the other version. All pictures were original or modified color ClipArt drawings. Objects were pre-tested, and modified as necessary, so that each generally elicited the same object name across subjects. These expected responses of the manipulated and non-manipulated objects were matched statistically to each other and between the two task versions on the frequency and length (number of syllables) of the singular (unmarked) name of each object, by including these variables as covariates in the analysis model (see Data Analysis). Objects were pseudo-randomized, separately within each task version, such that no more than four manipulated or non-manipulated objects were presented consecutively.

**Table 4:** Object naming task: items.

**Manipulated objects**	accordion, bow, chopsticks, comb, corkscrew, dart, drum, dustpan, eraser, faucet, fork, guitar, hammer, harp, iron, paintbrush, paperclip, pencil, pliers, saw, scissors, screwdriver, shovel, stapler, stethoscope, stopwatch, sword, telescope, toothbrush, umbrella, wallet, wrench, axe, bell, binoculars, broom, calculator, cane, clothespin, flashlight, flyswatter, gavel, hairbrush, ladle, lightbulb, lipstick, mailbox, match, megaphone, microphone, nutcracker, padlock, pen, piano, pitcher, pushpin, racquet, rake, razor, rolling pin, spoon, violin, whisk, whistle
**Non-manipulated objects**	ant, bat, beaver, chameleon, cheetah, chipmunk, eagle, elephant, flamingo, giraffe, gorilla, hummingbird, kangaroo, koala, lion, monkey, octopus, owl, panda, peacock, penguin, raccoon, scorpion, seahorse, skunk, spider, squirrel, swan, tiger, toucan, wolf, zebra, alligator, antelope, armadillo, bear, bee, bluejay, buffalo, cardinal, cobra, deer, dolphin, fox, frog, gazelle, grasshopper, hippopotamus, jellyfish, leopard, mink, moose, opossum, polar bear, puffin, ram, rhinoceros, seal, shark, starfish, stork, tortoise, turtle, whale

Subjects were instructed to name the depicted object as quickly and accurately as possible. After the instructions, they were given four practice items, which were presented in the same manner as the subsequent test items. Additionally, to acclimate the subject to the tasks, the test sequence began with 4 additional items (not counted in the 32 described above) that were excluded from analysis. Presentation procedure and parameters were identical to those of the past-tense production task, with the exception of the time-out period, which was 15 seconds (vs. 10 seconds in past-tense production). Response coding procedures were identical to those for the past-tense production task, except that responses were transcribed orthographically rather than phonemically. Responses were coded as correct only if they precisely matched the expected name of the object. For example, abbreviated responses (e.g., ‘*brella* for *umbrella*) or descriptions (*piano squeezer* for *accordion*) were considered incorrect, as were responses that contained phonological errors. This strict approach was taken to ensure comparability of response times across subjects for each item.

### Data Analysis

For each task, two mixed-effects regression models were constructed: one (with a logit-link function, for binary data) with accuracy of first responses as the dependent variable, and one with ln-transformed response times (RTs) of correct first responses as the dependent variable. RTs faster than 500 ms were discarded as likely errors (0.02% of valid RTs in both the past-tense production and object naming tasks) [29]. Subsequently, extreme outliers for each subject (responses whose RTs were more than 3.5 SDs from the given subject's mean) were removed for that subject (0.01% of responses in each task).

All models specified a three-way interaction between Session (1, 2), Treatment Group (DBS+ODT, ODT, Controls), and either Verb-type (regular, irregular for past tense production) or Object-type (manipulated, non-manipulated for object naming). For each model we first report the three-way interactions between each pair of treatment groups that we are interested in contrasting (DBS+ODT vs. Controls, ODT vs. Controls, and then direct comparisons between DBS+ODT and ODT). Each of these is followed by the two two-way interactions between Group and Session (either for each of the two verb-types or each the two object-types) that directly examine the predictions, as laid out in the Introduction. Any two-way interaction that was significant (α = 0.05) or approaching significance (<.10) was followed up with direct examinations of the underlying effects of interest, that is, potential differences between the two sessions (corresponding to on and off treatment for the patient groups) separately for each group and for each verb-type or object-type.

Several potentially confounding subject- and item-level factors were included as covariates in the models. Age (in years), years of education (starting from 1^st^ grade), and handedness (laterality quotients) were included as subject-level covariates. Item-level covariates for the past-tense production task were selected based on previous studies [29], [30]; see Table 5. Item-level covariates for object naming were log of order (analogous to that in Table 5), length (number of syllables in the singular form of the object name; included because word length predicts performance on single-word processing measures), and log of frequency of the singular form of the object name (analogous to stem frequency in Table 5).

**Table 5:** Item-level covariates included in the statistical models for the Past Tense Production task.

Variable	Explanation
Log of Order	The number of items presented prior to a given item. Including item order in the model allows one to account for variability attributable to presentation order (e.g., due to practice effects within the task). Order is likely to be more influential for earlier items, with order effects diminishing rapidly as participants become more comfortable with the task; therefore, as in previous studies [29], [30], the natural logarithm of item order was used.
Plosive	A binary variable describing whether the initial sound of the participant's expected response was a plosive. Included because this can affect computer-recorded response time measurements, since plosives tend to be detected faster than fricatives.
Fricative	A binary variable describing whether the initial sound of the participant's expected response was a fricative. See above.
Last Same	Whether or not the previously presented verb was of the same class (i.e., regular or irregular). Included because repeating a similar response (-*ed*-affixed) or producing a different type of response may affect RTs.
Last Real	Whether the previous verb was real or novel. Included because switching from a novel to a real response may affect processing time.
Log of Stem Frequency	Natural log of the frequency of the stem (unmarked) form of the verb. This and other frequency variables were based on frequency counts from two text-based corpora [29], [48]: (1) the Francis and Kucera counts [49], derived from 1 million words of text drawn from a variety of sources (“FK”); and (2) a frequency count extracted by a stochastic part-of-speech analyzer [50] from 44 million words of unedited Associated Press news wires between February and December 1988 (“AP”). Log of stem frequency was calculated as the natural logarithm of the sum of FK and AP plus 1. Included because word frequency can influence the time to produce words in tasks such as past-tense production and picture naming.
Log of PT Frequency	Natural log of the frequency of the correct past tense form of the verb [29], [48]. See just above for more information.
Stem Length	The number of phonemes in the stem, with diphthongs counted as one phoneme. Included because word length can predict performance on single-word processing measures.
Phonological Neighborhood	A measure of the frequency of phonologically similar and dissimilar verbs. Included to account for the influence of verbs with similar or dissimilar stem-past phonological transformations (e.g., the processing of *ring-rang* may be improved by *spring-sprang*, but weakened by *bring-brought*) (for more details, see [30]).
Noun-to-Verb ratio	An estimate of the likelihood that a given verb has been converted from a noun or into a noun, computed as the natural logarithm of the ratio of each stem's frequency as a noun to that form's frequency as a verb.

## Results

### Past Tense Production

**Figure 1:**
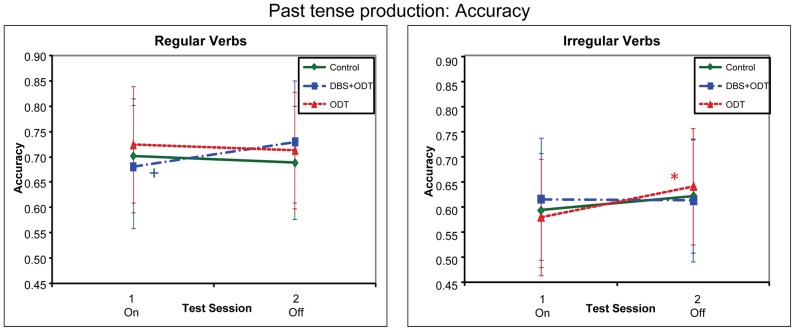
Accuracy for the Past Tense Production task. Accuracy for the three subject groups in the past tense production task, represented by adjusted means from the regression model. The DBS+ODT and ODT patients were on DBS/ODT during session 1 and off DBS/ODT during session 2. Significance levels are indicated only for session 1 vs. session 2 comparisons that are warranted by two-way interactions between group and session: +: p<.10; *: *p*≤.05; **: *p*≤.01; ***: *p*≤.001; ****: *p*≤.0001.

**Figure 2:**
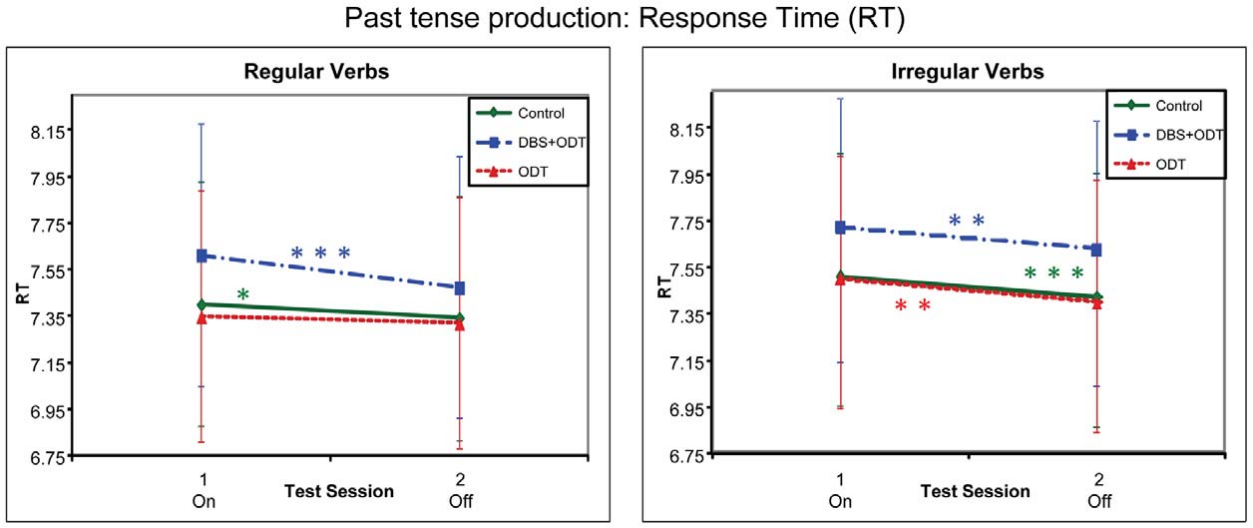
Response times (RTs) for the Past Tense Production task. Response times (ln-transformed RTs) for the three subject groups in the past tense production task, represented by adjusted means from the regression model. The DBS+ODT and ODT patients were on DBS/ODT during session 1 and off DBS/ODT during session 2. For significance levels, see Figure 1.

For past tense production, DBS+ODT patients showed worse performance (lower accuracy and slower response times) on than off treatment for regulars (unlike or relative to irregulars), as compared to both the controls and the ODT patients, who did not differ from each other across assessment sessions (Figures 1 and 2).

#### Accuracy

We first compared the DBS+ODT patients with controls. The three-way interaction between Treatment-group (DBS+ODT patients vs. controls), Session (1/on vs. 2/off), and Verb-type (regular vs. irregular) was significant (*B* = .02; *t*(2572) = 2.02, *p* = .04). In follow-up analyses, the two-way interaction between Group (i.e., Treatment-group) and Session was borderline significant for regulars (*B* = −.03; *t*(2572) = 1.88, *p* = .06) but not for irregulars (*B* = .02; *t*(2572) = .97, *p* = .33). This was explained by worse performance (lower accuracy) by the DBS+ODT patients for regulars on than off stimulation (*B* = −.05; *t*(2572) = −1.85, *p* = .06), with no difference between the two sessions for controls (*B* = .01; *t*(2572) = .67, *p* = .51), and no differences on irregulars between the sessions for either subject group (DBS+ODT patients: *B* = .003; *t*(2572) = .11, *p* = .91; controls: *B* = −.03; *t*(2572) = −1.44, *p* = .15).

Comparisons between the ODT patients and controls did not show this pattern. The three-way interaction between Group (ODT patients vs. controls), Session and Verb-type was not significant (*B* = −.01; *t*(2572) = −.63, *p* = .53). Similarly, neither of the two-way interactions between Group and Session, for either regulars or irregulars, approached significance (regulars: *B* = −.002; *t*(2572) = −.10, *p* = .92; irregulars: *B* = −.016; *t*(2572) = −1.01, *p* = .31).

Finally, we directly compared the DBS+ODT and ODT patients. The three-way interaction between Group (DBS+ODT vs. ODT patients), Session and Verb-type was significant (*B* = .03; *t*(2572) = 2.36, *p* = .02). This was explained by different patterns for regulars and irregulars. For regulars, the DBS+ODT patients showed worse performance on than off stimulation (see above), while the ODT patients showed no change from on to off treatment (*B* = .01; *t*(2572) = .39, *p* = .70). In contrast, for irregulars, the DBS+ODT patients showed no change between on and off stimulation (see above), while the ODT patients showed worse performance on than off treatment (*B* = −.06; *t*(2572) = −2.38, *p* = .02). Indeed, the two-way interaction between Group and Session showed opposite patterns (see *t* values) for regulars (*B* = −.03; *t*(2572) = −1.59, *p* = .11) and irregulars (*B* = .03; *t*(2572) = 1.76, *p* = .08). Note however that the ODT patients' pattern of worse performance at irregulars on than off treatment did not differ significantly from the pattern observed between session 1 and 2 for controls (see above), and thus does not suggest that medications alone lead to a relative deficit on irregulars.

#### Reaction Time

The comparison between DBS+ODT patients and controls showed a similar pattern for response times as for accuracy. Although the three-way interaction of Group (DBS+ODT patients vs. controls), Session and Verb-type was not significant (*B* = −.02; *t*(2174) = −1.26, *p* = .21), the two-way interaction between Group and Session was significant for regulars (*B* = .04; *t*(2174) = 2.10, *p* = .04) but not for irregulars (*B* = −.01; *t*(2174) = .27, *p* = .78). This was due to the fact that the DBS+ODT patients showed a larger deficit on as compared to off stimulation for regulars (*B* = .013; *t*(2174) = 4.43, *p*<.0001) than did the controls for the equivalent session 1 versus 2 (*B* = .06; *t*(2174) = 2.43, *p* = .02), while irregulars showed a similar decline for both groups (DBS+ODT: *B* = .10; *t*(2174) = 2.96, *p*<.01; controls: *B* = .09; *t*(2174) = 3.45, *p*<.001).

As with accuracy, the comparison between ODT patients and controls revealed no group differences between the sessions. Neither the three-way interaction between Group (ODT patients vs. controls), Session and Verb-type (*B* = .01; *t*(2174) = .82, *p* = .41), nor the two-way interactions between Group and Session (regular: *B* = −.01; *t*(2174) = −.74, *p* = .46; irregular: *B* = .01; *t*(2174) = .42, *p* = .67) were significant.

Finally, the direct comparison between DBS+ODT and ODT patients also showed a similar pattern to accuracy. The three-way interaction between Group (DBS+ODT vs. ODT patients), Session and Verb-type approached significance (*B* = −.03; *t*(2174) = −1.85, *p* = .07), and the two-way interaction between Group and Session was significant for regulars (*B* = .06; *t*(2174) = 2.51, *p* = .01) but not for irregulars (*B* = 00; *t*(2174) = −.13, *p* = .90). This was due to DBS+ODT patients showing a deficit on as compared to off stimulation for regulars (see above) while ODT patients showed no such difference (*B* = .03; *t*(2174) = .92, *p* = .36). In contrast, irregulars showed a similar decline for both groups (DBS+ODT: see above; ODT: *B* = .10; *t*(2174) = 3.17, *p*<.01).

### Object Naming

**Figure 3:**
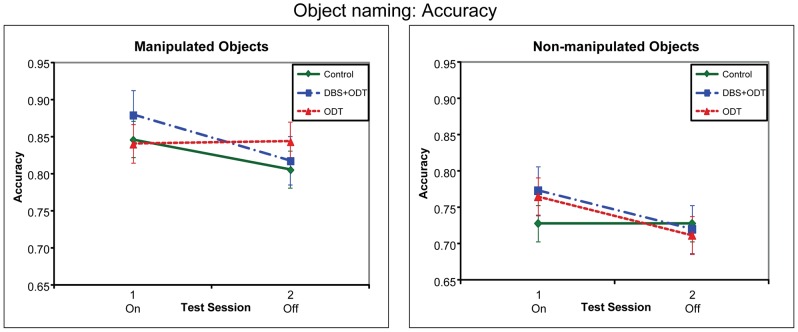
Accuracy for the Object Naming task. Accuracy for the three subject groups in the object naming task, represented by adjusted means from the regression model. The DBS+ODT and ODT patients were on DBS/ODT during session 1 and off DBS/ODT during session 2. For significance levels, see Figure 1.

**Figure 4:**
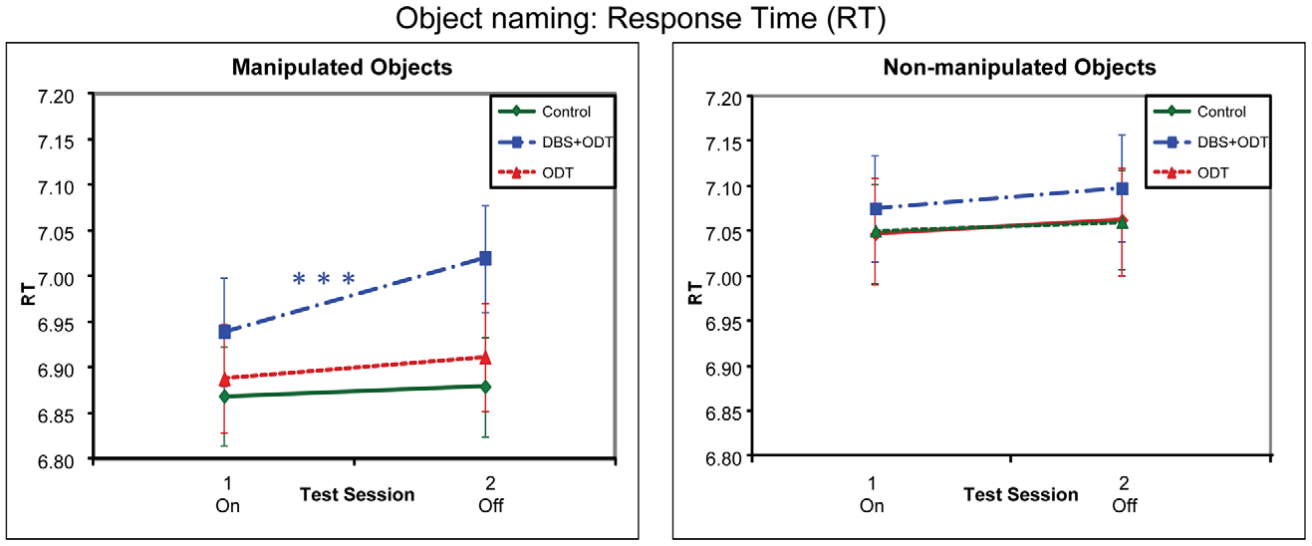
Response times (RTs) for the Object Naming task. Response times (ln-transformed RTs) for the three subject groups in the object naming task, represented by adjusted means from the regression model. The DBS+ODT and ODT patients were on DBS/ODT during session 1 and off DBS/ODT during session 2. For significance levels, see Figure 1.

For object naming, different patterns were obtained with accuracy and response times (Figures 3 and 4). For accuracy, none of the groups differed significantly from each other in the effect of treatment (i.e., session 1/on vs. 2/off), for either manipulated or non-manipulated objects. In contrast, for response times, DBS+ODT patients showed better (faster) performance on than off treatment for manipulated (but not non-manipulated) objects, as compared to both the controls and the ODT patients, who did not differ from each other across the sessions.

#### Accuracy

The comparison between DBS+ODT patients and controls revealed no group differences between the sessions for accuracy, with no three-way interaction between Group (DBS+ODT patients vs. controls), Session and Object-type (manipulated vs. non-manipulated) (*B* = .01; *t*(4308) = −.56, *p* = .58), nor any two-way interactions between Group and Session (manipulated: *B* = .01; *t*(4308) = .64, *p* = .52; non-manipulated: *B* = .03; *t*(4308) = 1.44, *p* = .15).

Similarly, in the comparison between ODT patients and controls, although the three-way interaction between Group (ODT patients vs. controls), Session and Object-type approached significance (*B* = .03; *t*(4308) = 1.81, *p* = .07), neither of the two-way Group by Session interactions were significant (manipulated: *B* = −.02; *t*(4308) = −.94, *p* = .35; non-manipulated: *B* = −.03; *t*(4308) = 1.62, *p* = .11).

Finally, there were again no session differences in the direct comparison between the DBS+ODT and ODT patients, with neither the three-way Group (DBS+ODT vs. ODT patients) by Session by Object-type (*B* = −.02; *t*(4308) = 01.13, *p* = .26) nor the two-way Group by Session interactions being significant (manipulated: *B* = .03; *t*(4308) = 1.42, *p* = .16; non-manipulated: *B* = 00; *t*(4308) = −.16, *p* = .88).

#### Reaction Time

The DBS+ODT patients showed a different pattern than the controls. Although the three-way Group (DBS+ODT, controls) by Session by Object-type was not significant (*B* = .02; *t*(3138) = 1.32, *p* = .19), the two-way interaction between Group and Session was significant for manipulated objects (*B* = −.03; *t*(3138) = −2.14, *p* = .03) but not for non-manipulated objects (*B* = −.003; *t*(3138) = −.20, *p* = .84). This was explained by the fact that for manipulated objects the DBS+ODT patients performed better (faster) on than off treatment (*B* = −.08; *t*(3138) = −3.28, *p* = .001), while the controls showed no difference between the two test sessions (*B* = −.01; *t*(3138) = −.65, *p* = .51). In contrast, non-manipulated items showed no differences between the sessions for either group (DBS+ODT patients: *B* = −.02; *t*(3138) = −.92, *p* = .36; controls: *B* = −.02; *t*(3138) = −.83, *p* = .41).

The comparison of ODT patients to controls did not show such group differences. The three-way interaction between Group (ODT patients vs. controls), Session, and Object-Type was not significant (*B* = .004; *t*(3138) = .34, *p* = .73); similarly, neither were the two interactions between Group and Session for either manipulated objects (*B* = −.003; *t*(3138) = −.16, *p* = .87) or non-manipulated objects (*B* = .01; *t*(3138) = .32, *p* = .75).

Finally, we compared the DBS+ODT and ODT patients directly. Although the three-way interaction between Group (DBS+ODT vs. ODT patients), Session and Object-type was not significant (*B* = .01; *t*(3138) = .87, *p* = .38), the two-way interactions between Group and Session differed between the two Object-types, approaching significance for manipulated objects (*B* = −.03; *t*(3138) = −1.78, *p* = .08) but not for non-manipulated objects (*B* = −.01; *t*(3138) = −.47, *p* = .64). For manipulated objects, the DBS+ODT patients showed better (faster) performance on than off treatment (see above), whereas the ODT patients showed no difference between the two treatment conditions (*B* = −.02; *t*(3138) = −.72, *p* = .47). In contrast, for non-manipulated objects, neither the DBS+ODT nor ODT patients differed between on and off treatment (DBS+ODT: see above; ODT: *B* = −.01; *t*(3138) = −.24, *p* = .81).

## Discussion

In brief, for past tense production, STN-DBS (with medications) led to lower accuracy at regulars but not irregulars, and to slower response times at regulars than irregulars, as compared to both healthy controls and patients with just medications, who did not differ from each other across assessment sessions. In contrast, for object naming, STN-DBS improved response times (with no changes in accuracy) for the naming of manipulated but not non-manipulated objects, as compared to the other two subject groups, who again did not differ from each other.

These results cannot be explained by various confounding factors, including subject-level variables such as age, education, handedness, or language history, or item-level variables such as frequency or word length, since these and other factors did not differ between groups or conditions or were controlled for statistically. The findings also cannot be explained by speed/accuracy trade-offs: for past-tense production, accuracy and response times showed the same performance patterns, while for object naming, accuracy showed no effects rather than the opposite effect as response times.

The finding that STN-DBS improved naming manipulated (but not non-manipulated) objects in early PD is consistent with previous studies showing that STN-DBS leads to motor improvements, as tested by physical motor outcomes, in advanced PD. The results suggest that previously observed STN-DBS related motor improvements in PD may be due at least partly to improvements in higher-level motor knowledge or processing, and not just to improvements of lower-level motor deficits such as tremor. Moreover, results from this well-controlled task demonstrate that observed motor improvements, or at least aspects related to higher-level motor knowledge and processing, do not seem to be due to confounding factors such as motivation. The findings further strengthen the evidence that naming manipulated objects depends on motor circuits. The results in this study particularly implicate the basal ganglia thalamocortical circuitry that is affected in early PD and the motor portions of this circuitry that are targeted in STN-DBS. It is not yet clear why the improvement in naming manipulated objects was found only with reaction time as the dependent variable, and not with accuracy. However, one possibility is simple ceiling effects in accuracy, since the DBS+ODT patients showed higher accuracy on than off treatment (see Figure 3), and on treatment they were quite close to ceiling.

The finding that STN-DBS impairs the production of regular but not irregular past-tenses was not predicted. Rather, we expected that STN-DBS would lead to improvements at regulars (but not irregulars). Crucially, the results differed from our predictions in the *direction* of the STN-DBS related effect on regulars, but *not* in which verb type (regulars) and thus which aspect of language (grammar) was affected. This further strengthens links between motor skills and grammatical processing (but not lexical processing), and links to the basal ganglia circuits affected in early PD and targeted by STN-DBS. However, it suggests a more complex relationship between grammar and motor function in the context of STN-DBS than had been anticipated, at least in early PD.

Indeed, the mechanisms underlying the differential impact of STN-DBS on motor and grammar function remain unclear. One possibility is that the pattern may be partly explained by the frequency of the stimulation in the current study (130 Hz), since it has previously been observed that high frequency STN-DBS (as was used here) can lead to motor improvement and cognitive decline, while low frequency STN-DBS enhances cognition but degrades motor function [44]. However, the finding that grammar but not lexical processing was negatively affected would still have to be explained. Another possibility is that the decline in performance on regulars is due to negative impacts of STN stimulation on other brain structures involved in grammar. For example, STN-DBS has been found to lead to PET activation changes in various cortical areas as well as in the ventral striatum, concomitant to degraded performance in a cognitive task [45]. However, the mechanisms underlying such changes remain unclear. A third possibility is that circuits (basal ganglia thalamocortical loops) that are anatomically close to those targeted by the stimulation suffer from some sort of competition or reorganization, though again the specific mechanisms would need to be determined. Further research on this issue seems warranted.

As discussed above, unlike for the object naming task, the results for the past-tense production task were not predicted. Moreover, they are only partly compatible with previous studies. On the one hand, they are not particularly inconsistent with previous studies of the impact of STN-DBS on lexical processing, since such studies have found that stimulation variously improves, worsens, or does not change performance on these tasks (see Introduction). On the other hand, the results are different from the two STN-DBS studies of syntax and morphology in advanced PD, which reported improvements on some measures, but no changes on others. One possibility is that some measures show improvements or no change in response to STN-DBS in PD, while others show declines. Another is that different patterns are found in advanced and early PD. A third possibility, however, is that carefully controlling for multiple task, subject- and item-level factors, as was done in the present study, might reveal the actual impact of STN-DBS on grammatical, as well as lexical, processing. Further studies should clarify these issues.

This study has several limitations. First, it is based on a relatively small sample size – though not particularly small compared to other studies of STN-DBS. Second, both the DBS+ODT and ODT patients were always tested on treatment before being tested off treatment. Given the protocol for the prolonged five day washout period, this order could not be easily reversed. Nevertheless, order effects were minimized by avoiding any item repetition between the sessions, in both tasks. Additionally, the direct comparison of on/off differences between subject groups (e.g., DBS+ODT patients to controls) precluded an explanation of more general order effects, unless they were specific to one of the groups. Third, although the DBS+ODT, ODT and control patients were directly or statistically matched on various factors, they might have differed on others (e.g., depression, general cognitive functioning). However, the design of the study, with the within- as well as between-subject and session examination of matched regular/irregular and manipulated/non-manipulated items, minimizes the likelihood of such factors explaining the observed findings. Fourth, and related to the previous point, UPDRS (Unified Parkinson's Disease Rating Scale) scores were not available, since the present study is part of an ongoing clinical trial, and these scores will not be made available until the termination of the trial. However, the strict inclusion and exclusion criteria (Table 1), including the fact that all PD patients were at stage II on the Hoehn and Yahr scale when off medication, ensures relative homogeneity within and between the two patient groups (DBS+ODT and ODT). Fifth, with respect to grammar, this study focused only on English regular past-tenses, and thus generalizations to grammar as a whole must be made with caution. Nevertheless, a large literature has linked the processing of English regular past-tenses to other aspects of grammar, including syntax [11], [46], suggesting that such a generalization may not be unwarranted. However, further studies are needed, ideally with the type of control of potentially confounding factors found in the present study. Sixth, it might seem surprising that the production of regular past-tenses was not impaired, as compared to controls, in the off state, given previous evidence that PD patients can show impairments at producing regular past-tenses [3]. However, those findings were observed in PD patients at a relatively advanced stage. Thus the lack of a deficit at regular past-tenses observed here is consistent with the more general pattern that cognitive impairments in PD occur at later stages of the disease.

In sum, this study examined the impact of STN-DBS on well-controlled language and motor tasks in patients with early PD, as compared to patients solely on medication, and healthy controls. STN-DBS impaired aspects of grammatical but not lexical processing, while improving aspects of motor function, as compared to both other subject groups, who did not differ from each other. The findings suggest that STN-DBS does indeed impact language, but may be specific in affecting aspects of grammatical but not lexical processing. The differential impact of STN-DBS on motor (improvement) and grammar (degradation) is surprising, and requires further investigation. The finding that aspects of both motor skills and grammar (but not lexicon) were affected by STN-DBS supports links between grammar and motor skills, and procedural memory more generally.

Although the STN-DBS related decline in grammar was found both in accuracy and response times, and thus is not trivial, the size of the observed effect does not seem likely to substantially impact the quality of life of the patients. Thus it still appears that DBS is a viable therapy for PD [7], as it effectively reduces motor symptoms and does not appear to have a major negative effect on language and other aspects of cognition. Nevertheless, the current results suggest the need for further research examining the impact of STN-DBS on language.
